# Supplementary material for: The Systems Biology Research Tool: evolvable open-source software
Source: BMC Syst Biol. 2008 Jun 29;2:55. doi: 10.1186/1752-0509-2-55 (PMC2446383; doi:10.1186/1752-0509-2-55)
Supplement: Additional file 1 — SBRT Archive. An archive of the current version of the Systems Biology Research Tool. [file 1752-0509-2-55-S1.zip › sbrt-1.4.0/doc/developers_guide/api/sbrt/kernel/math/algebra/TreeMatrix.html]

TreeMatrix


|  |  |  |
| --- | --- | --- |
| **Overview** \| **Package** \| **Class** \| **Use** \| **Tree** \| **Deprecated** \| **Index** \| **Help** | |  |
| **PREV CLASS**   NEXT CLASS | **FRAMES**    **NO FRAMES**     **All Classes** |  |
| SUMMARY: NESTED \| FIELD \| CONSTR \| METHOD | DETAIL: FIELD \| CONSTR \| METHOD |  |


---


## sbrt.kernel.math.algebra Class TreeMatrix<R extends java.lang.Comparable<? super R>,C extends java.lang.Comparable<? super C>>

```
java.lang.Object
  sbrt.kernel.math.algebra.AbstractMdMatrix<R,C>
      sbrt.kernel.math.algebra.AbstractSmdMatrix<R,C>
          sbrt.kernel.math.algebra.TreeMatrix<R,C>
```

**Type Parameters:**: `R` - the row type.: `C` - the column type.

**All Implemented Interfaces:**: DoubleMatrix<R,C>, DoubleMatrixBuilder<R,C>, Matrix<R,C,java.lang.Double>, MatrixBuilder<R,C,java.lang.Double>, SmdMatrix<R,C>, SparseDoubleMatrix<R,C>

---

``` public class TreeMatrix<R extends java.lang.Comparable<? super R>,C extends java.lang.Comparable<? super C>> extends AbstractSmdMatrix<R,C> ```

This class is a `java.util.TreeMap` based implementation of `SmdMatrix`.

**Author:**
:   This class was written and documented by Jeremiah Wright while in the Wagner lab.

**See Also:**: `RowOrTreeMatrix`, `HashMatrix`

---

**Constructor Summary**

`TreeMatrix()`
  
          Constructs an emtpy `TreeMatrix`.


| **Method Summary** | |
| --- | --- |
| `int` | `columns()`             Returns the number of columns in this matrix. |
| `java.util.TreeSet<C>` | `getColIndNzEl(java.lang.Object rowIndex)`             Returns the set of column indices whose elements are nonzero in the row corresponding to the provided index. |
| `TreeMatrix<R,C>` | `getColumn(java.lang.Object columnIndex)`             Returns the column corresponding to the provided column index. |
| `java.util.TreeSet<C>` | `getColumnIndices()`             Returns the set of column indices contained in this matrix. |
| `java.lang.Double` | `getElement(java.lang.Object rowIndex, java.lang.Object columnIndex)`             Returns the element of this matrix at the provided row and column indices. |
| `TreeMatrix<R,C>` | `getRow(java.lang.Object rowIndex)`             Returns the row corresponding to the provided row index. |
| `java.util.TreeSet<R>` | `getRowIndices()`             Returns the set of row indices contained in this matrix. |
| `java.util.TreeSet<R>` | `getRowIndNzEl(java.lang.Object columnIndex)`             Returns the set of row indices whose elements are nonzero in the column corresponding to the provided index. |
| `int` | `hashCode()`             Returns a content-based hash code for this matrix. |
| `boolean` | `isColumnIndex(java.lang.Object obj)`             Indicates if the provided object is a column index in this matrix. |
| `boolean` | `isRowIndex(java.lang.Object obj)`             Indicates if the provided object is a row index in this matrix. |
| `void` | `removeColumn(java.lang.Object columnIndex)`             Removes the column corresponding to the provided column index. |
| `void` | `removeRow(java.lang.Object rowIndex)`             Removes the row corresponding to the provided row index. |
| `int` | `rows()`             Returns the number of rows in this matrix. |
| `<X extends R,Y extends C,Z extends java.lang.Double> void` | `setElement(X rowIndex, Y columnIndex, Z value)`             Sets the element at the provided row and column indices to the provided double precision value. |

**Methods inherited from class sbrt.kernel.math.algebra.AbstractSmdMatrix**

`equals, setElement, setElements, setElements, setElements, toLinearCombs`

**Methods inherited from class sbrt.kernel.math.algebra.AbstractMdMatrix**

`isEmpty, setElements, toString`

**Methods inherited from class java.lang.Object**

`clone, finalize, getClass, notify, notifyAll, wait, wait, wait`

**Methods inherited from interface sbrt.kernel.math.algebra.DoubleMatrixBuilder**

`setElements`

**Methods inherited from interface sbrt.kernel.math.algebra.Matrix**

`isEmpty, toString`

**Methods inherited from interface sbrt.kernel.math.algebra.Matrix**

`isEmpty, toString`

**Methods inherited from interface sbrt.kernel.math.algebra.Matrix**

`isEmpty, toString`

**Constructor Detail**

### TreeMatrix

```
public TreeMatrix()
```

:   Constructs an emtpy `TreeMatrix`.


**Method Detail**

### setElement

```
public <X extends R,Y extends C,Z extends java.lang.Double> void setElement(X rowIndex,
                                                                            Y columnIndex,
                                                                            Z value)
```

:   Sets the element at the provided row and column indices
    to the provided double precision value.

    :   **Type Parameters:**: `X` - a type extending the primary row type.: `Y` - a type extending the primary column type.: `Z` - Double. **Parameters:**: `rowIndex` - the row index.: `columnIndex` - the column index.: `value` - the double precision element value.

---


### isRowIndex

```
public boolean isRowIndex(java.lang.Object obj)
```

:   Indicates if the provided object is a row index in
    this matrix.

    :   **Parameters:**: `obj` - the potential row index. **Returns:**: `true` if the provided object is a row index in this matrix; `false` otherwise.

---


### isColumnIndex

```
public boolean isColumnIndex(java.lang.Object obj)
```

:   Indicates if the provided object is a column index in
    this matrix.

    :   **Parameters:**: `obj` - the potential column index. **Returns:**: `true` if the provided object is a column index in this matrix; `false` otherwise.

---


### getRow

```
public TreeMatrix<R,C> getRow(java.lang.Object rowIndex)
```

:   Returns the row corresponding to the provided row index.
    A row is itself a 1 x n matrix, where n is the number of
    columns in this matrix.

    :   **Parameters:**: `rowIndex` - the index of the row to be returned. **Returns:**: the row corresponding to the provided row index.

---


### getColumn

```
public TreeMatrix<R,C> getColumn(java.lang.Object columnIndex)
```

:   Returns the column corresponding to the provided column index.
    A column is itself an m x 1 matrix, where m is the number of
    rows in this matrix.

    :   **Parameters:**: `columnIndex` - the index of the column to be returned. **Returns:**: the column corresponding to the provided column index.

---


### removeRow

```
public void removeRow(java.lang.Object rowIndex)
```

:   Removes the row corresponding to the provided
    row index.

    :   **Parameters:**: `rowIndex` - the index of the row to be removed.

---


### removeColumn

```
public void removeColumn(java.lang.Object columnIndex)
```

:   Removes the column corresponding to the provided
    column index.

    :   **Parameters:**: `columnIndex` - the index of the column to be removed.

---


### getElement

```
public java.lang.Double getElement(java.lang.Object rowIndex,
                                   java.lang.Object columnIndex)
```

:   Returns the element of this matrix at the provided
    row and column indices.

    :   **Parameters:**: `rowIndex` - the row index.: `columnIndex` - the column index. **Returns:**: the element of this matrix at the provided row and column indices.

---


### getColumnIndices

```
public java.util.TreeSet<C> getColumnIndices()
```

:   Returns the set of column indices contained in this
    matrix.

    :   **Returns:**: the set of column indices contained in this matrix.

---


### getRowIndices

```
public java.util.TreeSet<R> getRowIndices()
```

:   Returns the set of row indices contained in this
    matrix.

    :   **Returns:**: the set of row indices contained in this matrix.

---


### getRowIndNzEl

```
public java.util.TreeSet<R> getRowIndNzEl(java.lang.Object columnIndex)
```

:   Returns the set of row indices whose elements are nonzero
    in the column corresponding to the provided index.

    :   **Parameters:**: `columnIndex` - the column index. **Returns:**: the set of row indices whose elements are nonzero in the column corresponding to the provided index.

---


### getColIndNzEl

```
public java.util.TreeSet<C> getColIndNzEl(java.lang.Object rowIndex)
```

:   Returns the set of column indices whose elements are nonzero
    in the row corresponding to the provided index.

    :   **Parameters:**: `rowIndex` - the row index. **Returns:**: the set of column indices whose elements are nonzero in the row corresponding to the provided index.

---


### rows

```
public int rows()
```

:   Returns the number of rows in this matrix.

    :   **Returns:**: the number of rows in this matrix.

---


### columns

```
public int columns()
```

:   Returns the number of columns in this matrix.

    :   **Returns:**: the number of columns in this matrix.

---


### hashCode

```
public int hashCode()
```

:   Returns a content-based hash code for this matrix.

    :   **Specified by:**: `hashCode` in interface `Matrix<R extends java.lang.Comparable<? super R>,C extends java.lang.Comparable<? super C>,java.lang.Double>` **Overrides:**: `hashCode` in class `java.lang.Object`
    :   **Returns:**: a content-based hash code for this matrix.


---


|  |  |  |
| --- | --- | --- |
| **Overview** \| **Package** \| **Class** \| **Use** \| **Tree** \| **Deprecated** \| **Index** \| **Help** | |  |
| **PREV CLASS**   NEXT CLASS | **FRAMES**    **NO FRAMES**     **All Classes** |  |
| SUMMARY: NESTED \| FIELD \| CONSTR \| METHOD | DETAIL: FIELD \| CONSTR \| METHOD |  |


---
